# Transcranial Direct Current Stimulation Use in Warfighting: Benefits, Risks, and Future Prospects

**DOI:** 10.3389/fnhum.2019.00114

**Published:** 2019-04-18

**Authors:** Steven E. Davis, Glen A. Smith

**Affiliations:** ^1^Joint and Operational Analysis Division, Defence Science and Technology Group, Edinburgh, SA, Australia; ^2^Institute of Integrated and Intelligent Systems, Griffith University, Nathan, QLD, Australia

**Keywords:** biomedical enhancement, brain stimulation, cognitive enhancement, decision making, military, neuro-enhancement, transcranial direct current stimulation, tDCS

## Abstract

Transcranial direct current stimulation (tDCS) is a non-invasive brain stimulation technique which provides unique potential to directly improve human capability on a temporary, at needs, basis. The purpose of this paper is to consider the utility of tDCS through analysis of the potential risks and benefits in the context of defence service personnel. First, we look at the potential benefits, focusing primarily on warfighter survivability and enriching cognition quality in support of command and control. Second, we look at the potential detriments to tDCS military use, focusing on adverse effects, safety considerations, and risk. Third, we examine how the level of risk can be mitigated through military doctrine development focusing on safety parameters and exclusion criteria. Finally, we explore the future prospects of military tDCS use, particularly in terms of addressing gaps in the literature so that tDCS can be used ethically and efficaciously at the level of individual personnel.

## Introduction

The aspiration to increase cognitive and physical performance to foster protection from predators is nothing new. The boundaries of human performance have been tested and pushed through methods such as physical training, education, technology, and religion, for centuries (Levasseur-Moreau et al., 2013). More recently, evidence has emerged that aspects of cognition and motor performance can be enhanced through a technique called *transcranial direct current stimulation* (tDCS). tDCS is a non-invasive^1^ technique that modulates cortical tissue excitability, increasing or decreasing cerebral activity by applying a very low direct current (usually no more than 2 mA; 0.06 mA/cm^2^ over a 35 cm^2^ pad) from electrodes placed on the scalp. Approximately half of the current delivered to the head reaches and stimulates the brain (Dymond et al., 1975). Research has shown a wide range of cognitive effects, mainly dependent on the site and polarity of stimulation (the *montage*), and duration and pattern of current flow (the *protocol*). Generally brain function is increased under the positive anode; with either little effect or decreased function under the cathodal placement site (Jacobson et al., 2012). tDCS does not directly cause or block the firing of neurons; the immediate effects are thought to be a result of polarity-specific shifts in resting membrane potential (Sparing and Mottaghy, 2008). Enhancement effects of tDCS last beyond the duration of stimulation and can be detected up to 24 h after stimulation (depending on the montage, protocol, and cognitive aspect tested; Utz et al., 2010). The mechanism for these enduring effects are thought to be a result of long-term potentiation and long-term depression of neuronal synapses (Nitsche et al., 2008).

The utility of cognitive enhancement techniques have been examined by a number of authors, and non-invasive brain stimulation has been found to offer significant socioeconomic benefits, such as increasing productivity and growth (Buchanan, 2008), reducing inequality (Hai and Heckman, 2017), and encouraging social justice (Savulescu, 2006). However, these outcomes have little relevance in the context of enhancement for the military purposes considered in this paper. The utility of cognitive enhancement is different in military situations, as benefits must be weighed against the potential harms in circumstances where the ability to complete objectives safely and make better decisions can mean the difference between life and death (Wolfendale, 2008; McKinley et al., 2012).

The main purpose of this paper is to examine whether tDCS use can be ethical and efficacious in the enhancement of military personnel. First, an overview of the potential benefits of tDCS in terms of expanding warfighter-level capability and command and control decision making is provided. This is followed by a discussion of the potential harms the technology may produce for the military, including safety issues, adverse effects, risk of hitherto unobserved and undetected effects, risk of self-administration by military personnel, and how these issues represent a threat to military capability. Additionally, we examine how some of these risks can be mitigated. While it is not the purpose of the paper to propose a comprehensive set of guidelines covering military tDCS use, a number of recommendations are provided that can inform future doctrine development, including safety parameters and exclusion criteria. This section provides a frame of reference for the assumptions that are applied to the discussion of safety and risk. Finally, we identify the future prospects of tDCS use in the military, focusing on gaps in the existing literature which need to be addressed so that tDCS can be delivered ethically and efficaciously, and with the predictable and reliable individual-level effects that are required so that harm to military capability can be avoided.

## Value to Defence

National security involves a number of aspects of military defence, including warfighting, activity in preparation for war, and measures to prevent war (Paleri, 2008). While the core fundamental skills of soldiering have remained stable over time (movement, communication, and operating weapons; United States Department of the Army, 2005), the full gamut of defence activities has become increasingly complex. Seemingly straightforward skills must be coordinated and performed under great stress in dynamic, demanding, and unpredictable environments^2^. Conceptually, coordinated military action is achieved via three core elements of command and control: *capability*, which is utilised to achieve *intent*, given *awareness* (Lambert and Scholz, 2007). The rapid development of equipment, weapons, and systems means that warfighters operate in increasingly complex environments (Redmore, 2009), and high quality cognition is required in support of command and control (e.g., van Creveld, 1985; Storr, 2009). However, ever-increasing decision speed and data volume processing requirements are becoming progressively more mismatched with current human capabilities (United States Air Force Chief Scientist, 2010), and the ability to perform at the necessarily high level is hindered by the fact that every aspect of cognitive performance is degraded under the stress of combat conditions (Lieberman et al., 2005). In this section, the potential of tDCS to bridge this gap by supporting warfighter command and control is explored across the three core areas; achieving situational awareness, the formation (and communication) of intent, and capability.

The ability to make quick assessments of complex and rapidly changing situations is vital across all service branches and ranks, so maintaining situational awareness is an essential prerequisite of performing tasks in real-time (Tirre and Gugerty, 1999). A review conducted by Hartel et al. (1991) concluded that situational awareness deficits were the primary cause of military aviation related incidents, and Murray et al. (2010) attributed the majority of critical incidents involving naval Marine Corps infantry to failures of perception regarding consequential environmental or situational features. As presented in Table 1, there are signs that tDCS could improve a number of cognitive aspects relating to the attainment and maintenance of situation awareness. As well as the functioning of the perceptual senses (e.g., visual and auditory) themselves, attention and working memory are critical factors underlying in the acquisition of information in support of situational awareness (Ribeaux and Poppleton, 1978; Endsley, 1995).

**Table 1:** The potential of tDCS in the context of enriching situational awareness.

Targeted effect	Reference	Methodological characteristics	Outcome	Defence relevance
**PERCEPTUAL ABILITY**
Vision	Kraft et al., 2010	Anodal stimulation of visual cortex for 15 min at 1 mA current intensity.	Active group showed a significant increase in contrast sensitivity within 8 of the visual field compared with sham.	Visual perception is heavily relied on in search tasks, such as scanning for hostile threats.
Auditory perception	Tang and Hammond, 2013	Anodal auditory cortex stimulation at 1 mA for 20 min.	Active group exhibited degraded frequency discrimination compared with sham.	Hearing plays a critical role in maintaining situation awareness, such as in the detection and identification of signals (e.g., sniper localisation; Casali et al., 2011), and communication with team members (Garinther and Peters, 1990).
Tactile perception	Ragert et al., 2008	Anode stimulation of primary somatosensory cortex for 20 min with 1 mA current.	Improved somatosensory discrimination in active stimulation group compared with sham.	Tactile perception can be the primary source of situation awareness in conditions where visibility is restricted (such as underwater work or in heavy smoke). Tactile sensitivity is also essential to complete tasks that require very fine manual precision (such as knot tying).
Navigation	Hampstead et al., 2014	Anodal stimulation over parietal midline coupled with cathodal stimulation over the superior frontal gyrus of 2 mA for 20 min.	Active group showed increase in activation and connectivity in spatial navigation regions compared with reverse polarity group.	While modern forces predominantly rely on Global Navigation Satellite System receivers for monitoring position awareness (of both friendly and enemy units and installations), the ability to navigate without the aid of sophisticated technology remains critical to soldiering (United States Department of the Army, 2005).
**ATTENTION**
Executive attention	Coffman et al., 2012	Anodal stimulation over right inferior frontal cortex for 30 min.	2 mA group showed increase in alerting measures of attentional networks compared with 1 mA group.	Attentional flexibility is required to productively allocate finite cognitive resources (Anderson, 2005). Situational


Selective attention	Clark et al., 2012	Anodal stimulation of 2 mA over right inferior frontal scalp for 30 min.	Active group identified more correct targets, experienced fewer false alarms, and completing the task quicker than sham group.	awareness cannot be achieved without the ability to retain and access relevant information (Gutzwiller and Clegg, 2013).
Spatial attention	Loftus and Nicholls, 2012	Anodal stimulation (1 mA) of left posterior cingulated cortex for 20 min.	Active group demonstrated reduction in pseudoneglect compared with sham and cathodal groups.	
Sustained attention (vigilance)	Nelson et al., 2014	Anodal stimulation over left dorsolateral prefrontal cortex coupled with cathodal stimulation over right dorsolateral prefrontal cortex at 1 mA intensity for 10 min.	Active group displayed enhanced accuracy but slower reaction time when compared with sham stimulation group.	
Visual search	Binney et al., 2018	Anodal stimulation of middle temporal gyrus with cathodal frontotemporal stimulation for 20 min at 2 mA.	Stimulation produced longer initial and cumulative visual fixations on key semantic features.	
**MEMORY**
Declarative memory	Marshall et al., 2004	Intermittent (15 s on, 15 s off) anodal stimulation (0.26 mA) of F3 and F4^1^ brain areas during slow-wave sleep over a 30 min period.	Stimulation during sleep increased the retention of word pairs when compared to sham stimulation or active stimulation while awake.	Memory ability is particularly associated with performance on a number of tasks where situation awareness is imperative, including fighter piloting (Carretta et al., 1996), dynamic decision-making (Gonzalez and Wimisberg, 2007), air traffic controlling (Gronlund et al., 1997), and point-shooting (Kleider et al., 2009).
Long-term memory	Javadi and Cheng, 2012	Anodal stimulation of left dorsolateral prefrontal cortex for 20 min with a current of 1.5 mA.	Active stimulation group recalled significantly more words than cathodal and sham groups.	
Working memory^2^	Gladwin et al., 2012	Anodal stimulation of 1 mA for 10 min over left dorsolateral prefrontal cortex.	Participants in active group exhibited faster recall than sham group.	

*^*1*^Using the 10–20 electrode system (see Klem et al., 1999).*

*^*2*^Note that as well as being selectively enhanced, there is evidence that with different electrode placements, working memory can be selectively impaired (e.g., Berryhill et al., 2010).*

Coordinated action must be achieved through the establishment of common intent (Pigeau and McCann, 2000). Commanders are tasked with making sound judgments and decisions while facing complex problems in unpredictable situations (Crabbe, 2000), so highly sophisticated cognitive skills are required to support commanders’ ability to form achievable intent (Allen and Gerras, 2009). Increasingly, decision-making authority is dispersed to lower ranks as increasing warfare dynamism requires a higher level of decision making immediacy to exploit time-limited tactical opportunities (i.e., “mission command”; Joint Chiefs of Staff, 2012; Australian Army, 2017). This shift towards decentralisation of command initiative means that the levels of command are unavoidably blurred (Pigeau and McCann, 2000), so the requirement to make sound decision-making is increasingly applicable down the chain. tDCS has shown the potential to improve a number of aspects of cognition relevant to the formulation of intent, including planning, problem solving, probabilistic assessment, and acceptance of risk^3^. Once intent is reached, it must be shared through effective and efficient communication in order to achieve coordinated action (Pigeau and McCann, 2000). As shown in Table 2, tDCS has shown potential in both the formation and communication of intent.

**Table 2:** The potential of tDCS in the context of forming and communicating intent.

Targeted effect	Reference	Methodological characteristics	Outcome
G**REASONING**
Cognitive bias mitigation	Forcano et al., 2018	Literature review article.	Numerous studies have shown tDCS to increase the effects of cognitive training in areas like reducing food cravings and improving diet habits.
	McIntire et al., 2017a	Anodal stimulation of left dorsolateral prefrontal cortex.	Anodal stimulation significantly diminished the benefit of the cognitive bias mitigation training compared with the sham stimulation group.^1^
Moral judgment	Ye et al., 2015	Simultaneous 2 mA anodal and cathodal stimulation of the right and left temporoparietal junctions for 20 min (participants began the task after 15 min).	Group receiving active stimulation judged intentional harm scenarios as less morally permissible than both sham and reverse polarity stimulation groups.
Planning	Dockery et al., 2009	Two configurations were examined (1 mA, 15 min): (1) Anodal left dorsolateral prefrontal cortex stimulation. (2) Cathodal left dorsolateral prefrontal cortex stimulation.	Group one completed the task faster than the sham group, while group two completed the task more accurately than the sham group.
	Heinze et al., 2014	Concurrent anodal stimulation of right dorsolateral prefrontal cortex and cathodal stimulation of right dorsolateral prefrontal cortex for 15 min with 1 mA current (the task commenced 5 min into stimulation).	Active group presented improved planning performance (decreased initial thinking time and gaze shifts) compared with reverse polarity group.
Probabilistic assessment	Hecht et al., 2010	Anodal stimulation of left dorsolateral prefrontal cortex coupled with cathodal right dorsolateral prefrontal cortex for the duration of five-block experiment (∼22 min) at 2 mA intensity.	The active stimulation group were quicker to select the most frequent alternative compared with control groups.
Problem solving	Chi and Snyder, 2011	Cathodal stimulation over the left anterior temporal lobe coupled with anodal stimulation over right anterior temporal lobe at 1.6 mA intensity. Stimulation began 5 min before testing, and continued until completion (up to 17 min).	Active stimulation group were three times more likely to solve an insight problem-solving task than the sham stimulation group.
Deceptive capabilities^2^	Karim et al., 2010	Cathodal stimulation of anterior prefrontal cortex at 1 mA for 13 min.	Active stimulation group were better at deceiving than participants in sham group.
G**COMMUNICATION**
Communication skills	Schülke and Straube, 2017	Anodal and cathodal stimulation (1.5 mA for 10 min) to the frontal, parietal and frontoparietal cortices.	Anodal stimulation group exhibited decreased reaction times on metaphoric co-verbal gesture task compared with cathodal group.
Empathy^3^	Wang et al., 2014	Anodal stimulation of left dorsolateral prefrontal cortex for 5 min with 2 mA current.	Pain empathy was enhanced in stimulation group compared with sham group.

*^*1*^Despite the direction of the relationship, the study shows that stimulation can affect cognitive bias mitigation. tDCS may be beneficial with alternate stimulation montages/protocols when additional research is conducted in this area. ^*2*^Deliberate misleading of adversarial decision makers is regularly employed to deter unfavourable hostile actions (or encourage favourable actions) to increase the likelihood of friendly mission success (Joint Chiefs of Staff, 2012). ^*3*^In volatile and unstable environments, empathy is an essential attribute for warfighters to relate to a diverse range of people with different ethnic, cultural, religious, linguistic, and/or socioeconomic backgrounds.*

The third component of the trinity of elements associated with achieving coordinated action (Lambert and Scholz, 2007) is capability. Warfighters need sufficient and specific levels of mental and physical preparedness to be capable of carrying out commanders’ intent (Royal Australian Air Force, 2017; Australian Army, 2018), and tDCS shows promise in a number of areas related to the capability of individual warfighters, including physical competence, training and education, and resilience. The areas of tDCS which show promise in assisting personnel to increase their capability to complete demanding undertakings while managing physical and psychological stressors are presented in Table 3.

**Table 3:** The potential of tDCS in readying military personnel for the physical and psychological requirements and stressors of warfighting.

Targeted effect	Reference	Methodological characteristics	Outcome	Defence relevance
**PHYSICAL PERFORMANCE**
Motor precision	Pavlova et al., 2013	Anodal stimulation of left dorsal premotor cortex.	Active group showed significantly improved fine motor control compared with sham group.	The United States Army has emphasised physical ability as a key element in military training since the lack of physical preparedness was found to be a factor in the loss at the Battle of Osan (Cannon, 1988; Thomas et al., 2004). Physical performance depends on neurological factors as well as skeletal muscle attributes (Noakes, 2011, 2012), so centrally-acting agents have been shown to modify athletic performance (Machado et al., 2018). tDCS has shown potential in a number of areas which may be able to enhance the physical capability of warfighters, although findings in this area have hitherto been mixed. See Machado et al. (2018) and Holgado et al. (2019) for reviews of the literature on the effects of tDCS on physical performance.
Motor strength	Tanaka et al., 2009	Anodal stimulation over leg area of motor cortex for 10 min at 2 mA.	Active group displayed greater pinch force than sham and reverse polarity groups.	
Muscle endurance	Cogiamanian et al., 2007	Anodal stimulation of motor cortex for 10 min at 1.5 mA.	Anodal group exhibited decreased rate of muscle fatigue compared with cathodal and no stimulation groups.	
Whole-body exercise	Vitor-Costa et al., 2015	Anodal stimulation of primary motor cortex for 13 min at 2 mA.	Performance on a constant-load cycling task was improved compared with reverse polarity and sham stimulation.	
Increased inhibitory control	Ditye et al., 2012	Anodal stimulation of right inferior frontal gyrus at 1.5 mA for 15 min.	Neuromodulation combined with training produced greater improvement in behavioural inhibition performance than the training alone.	Deployed personnel carry live ammunition, so a high level of discipline is required to ensure that soldiers remain in control of their weapon at all times (Redmore, 2009).
	Jacobson et al., 2011	Unilateral anodal stimulation at 1 mA for 10 min over right inferior frontal gyrus.	Active group showed significantly improved response inhibition compared with both sham and alternative stimulation site (right angular gyrus).	
**TRAINING/EDUCATION**
Associative learning	Flöel et al., 2008	Anodal stimulation of 1 mA over left lateral sulcus for 20 min.	Active group displayed faster associative learning than sham group.	Education and training play a significant role in all aspects of competency development. This includes the specific soldiering skills of movement, using a weapon, and communicating, as well as more general but equally important skills such as using technical devices, learning to manage food and nutrition, and other aspects of individual performance (Hicks, 1972; British Army, 1981; United States Department of the Army, 2005). The potential of tDCS to augment training and learning is particularly promising in terms of military utility. Research directed at increasing workforce efficiencies through augmentation of human
Categorisation learning	Lupyan et al., 2012	Anodal stimulation of 1.5 mA for 20 min of left inferior frontal cortex.	Active group increased low-dimensional categorisation learning^1^ and decreased high dimensional categorisation^2^ compared with cathodal and sham groups.	
Training time	McKinley et al., 2013	Anode stimulation of the right inferior frontal gyrus at 2 mA for 30 min.	Following 90 min of training on a target search and identification task, participants in the active stimulation group achieved 25% higher visual search accuracy than reverse polarity and training only groups.	
Motor learning	Nitsche et al., 2003b	Anodal stimulation (1 mA for 15 min) of primary motor cortex in target group; anodal premotor and prefrontal stimulation in two control groups.	Target group were significantly faster at executing implicitly learned sequences using contralateral hand than participants in either control group.	
Skill acquisition	Fujiyama et al., 2017	Cathodal stimulation of primary motor cortex for 10 min prior to skill training, and anodal stimulation for 20 min during training.	Group who received pre-training cathodal stimulation later displayed greater skill improvement than pre-training sham group.	performance was identified as the second highest of nineteen key priorities facing the United States Air Force over the next decade (United States Air Force Chief Scientist, 2010). The potential of tDCS to reduce training time and/or increase retention could provide both fiscal and capability improvements.
**RESILIENCE**
Ruminative thinking inhibition	de Raedt et al., 2017	Anodal stimulation of left dorsolateral prefrontal cortex for 20 min at 1.5 mA.	Active group exhibited a significant decrease in momentary ruminative self-referential thinking compared with sham group.	By its nature, warfighting involves high levels of danger, challenge, adversity, and risk (Gilmore, 2016). In order to complete missions successfully and maintain physical and psychological health, personnel must be highly resilient (Davis et al., 2009; Salehinejad et al., 2017). As well as increasing the risk of post-traumatic stress disorder (Godwin and Kreutzer, 2013), low resilience and poorly managed stress and anxiety can adversely affect situational awareness and decision making (e.g., Kleider et al., 2009; Wallenius et al., 2009), which can lead to poor military outcomes (including casualties and/or mission failure; Gilmore, 2016).
Stress resilience	de Jesus and Gonçalves, 2012	Anodal stimulation of left dorsolateral prefrontal cortex for 20 min at 2 mA.	Active group increased parasympathetic activity and decreased sympathetic activity compared with sham group.	
Trait resilience	Salehinejad et al., 2017	Anodal tDCS of the left dorsolateral prefrontal cortex for 15 min at 1 mA.	Active stimulation group showed greater improvement in trait resiliency than sham group.	
Fatigue reduction	McIntire et al., 2017b	Dorsolateral prefrontal cortex stimulation for 30 min at 2 mA.	Active group reported more vigour, less fatigue, better mood, and less boredom during 36 h sustained wakefulness session, compared to both a sham group and caffeine taking group.	Sleep deprivation is a practically unavoidable consequence of conducting prolonged, demanding, and high-tempo operations, and inadequately managed fatigue has a number of can have a number of consequences such as physical and psychological stress, poor decision making, diminished morale, and the inability to sustain the required level of battle proficiency (Murphy, 2002).
Hunger resistance	Goldman et al., 2011	Simultaneous anodal and cathodal stimulation of right and left prefrontal cortices (respectively) for 20 min at 2 mA.	Active stimulation led to greater reduction in food cravings than sham stimulation.	The prevalence of obesity is increasing globally (Afshin et al., 2017), and the rates of overweight military personnel is increasing likewise (Smith et al., 2012). This could impact the ability of warfighters to meet the physical standards required to effectively perform their duties.
Pain management	Aslaksen et al., 2014	Anodal stimulation of primary motor cortex for 7 min at 2 mA.	Stimulation produced analgesic effect on high-intensity heat pain.	While unlikely to prove practical in terms of managing pain on the battlefield, it is worth noting that under-managed acute pain can often evolve into chronic pain, as well as increasing the risk of secondary ailments such as deep vein thrombosis or myocardial ischemia (Vallerand et al., 2015). The potential of tDCS to support military provided medical care in the management of pain may be worth investigating further.

*^*1*^Low dimensional categorisation tasks involve classification of items based on concrete components (like shape and colour). ^*2*^High dimensional categorisation tasks involve classification of items based on thematic categories (e.g., “objects that hold water”).*

## Risks

There has been a great deal of research on the safety of tDCS which indicates that the technique is broadly safe, and does not cause permanent or severe damage or discomfort when used according to appropriate guidelines (such as those suggested in the *Risk Mitigation* section of the present paper). In a meta-analysis conducted by Brunoni et al. (2011) which examined the effects of tDCS in humans in every available paper from 1998 to 2010, no serious or ongoing side effects were reported. However, tDCS is not entirely risk-free. The technique is still relatively new, and has been associated with mild and transient discomfort in some people, including reported tingling, itching, and burning sensations under the electrodes.

The most common adverse effect of tDCS is a mild burning/itching sensation at the point of contact with the electrodes (Been et al., 2007). Light itching under the stimulation electrodes occurs in approximately 30% of cases (Poreisz et al., 2007). Although sensory side effects are reasonably common, severity is typically low (Kessler et al., 2012). Falcone et al. (2012) conducted a study in which participants rated each common sensation on a 10 point Likert scale, where 1 indicated no sensation and 10 indicated extreme sensation. Mean scores in the active condition for itching, heat, and tingling were 1.81, 1.44, and 2.51, respectively. The most serious adverse effects were two reported cases of skin lesions following repeated tDCS sessions (Palm et al., 2008; Frank et al., 2010). The authors suggested that an accumulation of impurities from tap water on the electrodes caused the reaction. The risk of both skin lesions and itching/burning sensations can be minimised by soaking the connecting sponges in sodium chloride (saline) solution rather than water before commencement of stimulation (DaSilva et al., 2011).

Mild headaches are sometimes observed. They are generally brief and are more commonly reported by frequent headache sufferers (e.g., 3.9% in healthy subjects vs. 11.1% in migraine patients; Poreisz et al., 2007). Some studies have reported a higher incidence of headaches in sham conditions than active stimulation conditions (14.8% active vs. 16.2% sham; Brunoni et al., 2011), though these findings have not been consistent across studies (e.g., Kessler et al., 2012). The extent to which headaches are a direct effect of tDCS is unclear, with secondary factors (such as pressure from the strap that keeps the electrodes in place) likely also playing a role. Nausea has been also experienced within 2 h of tDCS, though such reports are infrequent (less than 3% of participants; Poreisz et al., 2007).

Fatigue is sometimes reported by participants following tDCS studies. For example, Brunoni et al. (2011) found that a small to moderate level of fatigue was experienced by 35.3% of participants. However, it is not clear whether fatigue is directly caused by the stimulation itself, or is the result of a secondary cause such as an increase in concentration to complete the tasks that are often completed in conjunction with tDCS (i.e., what is being measured by the particular study; often problem solving related tasks), or by an increase in nervous energy experienced by participants undergoing an unfamiliar technique involving wires, straps, and electrodes. Conversely, there is evidence that particular stimulation montages can in fact reduce fatigue (e.g., McIntire et al., 2017b).

Overall, transient minor discomfort is present in about 10.4–17.7% of participants (Poreisz et al., 2007; Brunoni et al., 2011); not acute enough to cause subjects to request early termination of stimulation. Nevertheless, all documented adverse effects need to be considered and managed in any military tDCS programme. It is the view of the current authors that since all documented harms are either minor or can be mitigated, there is no case for dismissing military tDCS use on the basis of reported adverse effects. However, the potential for delayed and/or unknown harms also need to be considered.

Given the relative recency of tDCS, it is possible that hitherto unencountered effects could later emerge in some people. Long-term risk falls broadly into two categories: delayed effects that emerge after short term use, and cumulative harm following prolonged use. Although tDCS has only become heavily researched in recent years, the possibility of delayed adverse effects is somewhat mitigated by the fact that various forms of direct current have been used for over 100 years (e.g., for the treatment of melancholy in 1804; Fox, 2011), yet no delayed effects have been documented to date. tDCS has been heavily studied over the last decade, and although it is not clear whether it may cause negative consequences that take more than 10 years to materialise, there is no evidence to suggest this is the case. Risks associated with prolonged use are not well understood either. Given evidence that repetitive daily tDCS can lead to lasting long-term targeted changes (Agnew and McCreery, 1987), it follows that repetitive tDCS could cause long-lasting detrimental effects. Overall, little is known about either form of long-term risk, but there is currently no evidence that tDCS causes long-term damage when used within appropriate guidelines.

It is currently unclear how cognitive functioning in non-target areas is effected by tDCS. The precise path the electrical current follows is not fully understood (McKinley et al., 2012) and cannot easily be controlled, so brain regions interconnected to the target area under the electrodes could receive a portion of the current (Sadleir et al., 2010). Subsequent activations and deactivations associated with electrical stimulation can even occur in remote locations across both cerebral hemispheres (Utz et al., 2010). Moreover, activity in areas of the brain not directly under the electrodes may be reduced, creating an overall equilibrium between the cognitive areas of interest and other cognitive areas (Hilgetag et al., 2001). While most tDCS studies report enhancement of function, measures are generally limited to a specific area of cognition relating to the identified functional specialisation of the underlying brain region. Brem et al. (2014) describe a “net zero-sum model” in which the principle of conservation of energy governs the allocation of brain resources. This represents a risk in the military, where the possibility of unpredictable cognitive or physical deficits could make already dangerous military situations even riskier. More research employing multiple assessments across various areas of cognition needs to be conducted.

It is also unknown whether tDCS has the potential to be addictive. Because the neurotransmitter dopamine is involved in both the reward-related processes associated with addiction (Nutt et al., 2015) and high level cognitive functioning (e.g., Nieoullon, 2002; Cools, 2011), many forms of cognitive enhancement have been found to be addictive (such as central nervous system stimulants; Dackis and Gold, 2008). There is some limited evidence associating tDCS with altered dopamine levels (e.g., Fonteneau et al., 2018), but it is unclear whether this association is a result of direct dopaminergic modulation (such as is the case with stimulants like amphetamines and modafinil; Fleckenstein et al., 2007; Volkow et al., 2009). The size of dopaminergic effect^4^ appears to be more aligned with indirect dopamine concentrations^5^ than with drugs that directly increase dopamine levels in the reward system^6^, so the effect of tDCS on dopamine levels appears subtle. No issues with addiction have hitherto been reported in the literature, and the enduring effects of tDCS across multiple sessions appear more disposed towards accumulation of effect than development of tolerance (Alonzo et al., 2012). While the extant evidence does not suggest addictive potential, addiction is a multidimensional occurrence that cannot be predicted by any single neurological (Sinha, 2011) or psychosocial (Nation and Heflinger, 2006) variable, so the possibility of risk cannot be discarded. More research is required in this area; particularly given the significantly increased likelihood towards developing addiction observed in military populations (e.g., Lande et al., 2008; Kelsall et al., 2015).

The fact that techniques like tDCS are described as ‘non-invasive’ may produce the misleading impression that improper administration is less problematic or dangerous than overtly internal forms of enhancement^7^. tDCS has been described in the popular press, and there are several do-it-yourself (DIY) websites describing how to build (poor quality) tDCS apparatuses. All that is needed to build a device at home is a 9v battery, around $40 (US) of electronic parts, and instructions available through forums and dedicated websites (Fitz and Reiner, 2015). DIY brain stimulation is becoming so common that governmental bodies have begun issuing warnings on the dangers of using unapproved devices purchased through the internet (e.g., California Department of Public Health, 2013). It can therefore be expected that military personnel may be interested in self-administration using machines made at home or purchased online. This type of use carries a greater level of risk due to the lack of essential safety features, component redundancy, safe administration guidelines, and expert knowledge of appropriate montage and protocol setup that would be present in professional settings. Anecdotes of serious and permanent side effects have been described in online discussion forums, even including loss of consciousness (Dcminsf, 2014; Packmanta, 2014). Self-administration increases the risk of inappropriate or dangerous montages. For example, in one extreme (but isolated) case, direct current was used to stimulate the brainstem, leading to a number of serious (albeit temporary) adverse effects including disturbed breathing, speech arrest and psychosis (Lippold and Redfearn, 1964). Additionally, self-administration could cause additional risk in military situations where unanticipated or inconsistent effects could have considerable consequences for the capability of warfighters and their ability to properly complete their mission objectives.

## Risk Mitigation

It is important to note that with the exception of DIY tDCS, the risks and safety issues discussed in the previous section are underpinned by assumptions about how tDCS is delivered. As well as providing a frame of reference to make these assumptions explicit, this section serves the second purpose of providing guidance on how tDCS guidelines ought to be developed to ensure that tDCS can be delivered safely and efficaciously in military environments. Specifically, this section explores general safety procedures and screening criteria for personnel in which tDCS may be contraindicated.

While tDCS is generally safe, it has been found to cause permanent damage in animal studies when high levels of current were used. There is a risk of causing tissue damage as the electrical stimulation begins to heat brain tissue (Agnew and McCreery, 1987). In a study involving rats, Liebetanz et al. (2009) found that brain lesions formed when current densities exceeded 143 A/m^2^. Studies and treatment involving humans typically use current less than 0.0057 A/m^2^ (2 mA through a 35 cm^2^ pad). While there is some variation in the current limits recommended by key papers on the topic, most recommendations fall within the 1.5–3 mA range. For military operational stimulation, we recommend a current density limit of 2 mA (four orders of magnitude less than the current densities shown to cause lesions in rats). For military research purposes however, a limit of 1.5 mA may be more appropriate to ensure adequate participant blinding (Hummel and Cohen, 2005), since some participants are able to distinguish between active and sham stimulation at intensities at or above 2 mA (O’Connell et al., 2012). Medically certified tDCS devices are fitted with current limiting features, preventing accidental exposure to damaging current levels, and should be the only devices considered for military use. tDCS devices should also undergo regular electrical verification and validation to ensure the equipment is calibrated correctly, and continues to meet safety requirements and specifications.

Several papers within the current body of relevant literature have proposed universal tDCS safety standards and limits. For example, see Nitsche et al. (2003a) or Iyer et al. (2005) for discussion on general safety and limits, and DaSilva et al. (2011)^8^ for direct procedural guidelines on employing standardised tDCS protocol, including materials, measurements, skin preparation, electrode positioning, tDCS administration, and evaluation. There is currently no case for procedural guidelines covering stimulation in military contexts to deviate substantially from general guidelines, so we recommend that any military use of tDCS is consistent with guidelines and recommendations stipulated in the literature cited above.

In some studies, participants experienced temporary mood, personality and behavioural changes following tDCS. Several stimulation montages have been found to modulate relevant neural systems so as to affect a number of key aspects associated with social interaction and empathy, including resonance, self-other discrimination, and mentalisation (Hétu et al., 2012). Additionally, tDCS is known to have other effects on personality and behaviour in areas such as emotion (e.g., emotional aspects associated with pain; Maeoka et al., 2012), honesty (e.g., lying and deception; Priori et al., 2008), mood (e.g., depression/sadness; Ferrucci et al., 2009), comfort with risk (e.g., risk-taking and reward seeking behaviour; Fecteau et al., 2007), and even personal ethics and morality (e.g., judgments on the moral permissibility of attempted harms; Young et al., 2010). Influencing mood, personality and behaviour is not trivial. As put by Hamilton et al. (2011, p. 189), “tDCS can transiently alter an individual’s understanding of and relationship to others in ways that touch directly on the neural basis of ethical and moral thought and behaviour”. While it is worth noting that this type of stimulation could potentially be used beneficially in some circumstances, there are a number of ethical issues that need to be addressed that go beyond the intended scope of this paper. Regardless of ethical considerations, an unanticipated or unpredictable alteration of personality or behaviour represents a tangible threat to capability. Our current recommendation for military-based tDCS research, therefore, is that ongoing mood and personality should be closely monitored and reported, particularly in research areas involving novel and underreported electrode montages.

Another important factor to ensure safe delivery of tDCS concerns the personnel that are selected. tDCS affects participants with particular psychological, mood, and physiological conditions differently to healthy participants (Poreisz et al., 2007). Stringent exclusion criteria therefore need to be set out in military guidelines, covering both research and operational tDCS applications. Recommendations on exclusion criteria differ across key papers, and there is some variation on which criteria should be included. In the context of warfighting, the consequences of unpredictable or adverse outcomes go beyond the general human research principle of avoiding unnecessary distress and injury. In circumstances where adverse effects could prevent personnel from being able to perform their role to their full ability, the capability of the individual or unit may be at risk. In cases where contradictory recommendations exist, the more conservative approach should therefore generally be adopted.

Personnel should be excluded from tDCS if they have suffered from any skin diseases, have metallic implants near the electrodes, or suffer from any unstable medical condition or illness that could increase the risks of stimulation (Nitsche et al., 2003a). This includes (but is not limited to) neurological diseases, chronic eczema, and signs of epilepsy. Personnel taking medication known to affect the central nervous system should also be excluded, including narcoleptic and antiepileptic drugs, antidepressants, benzodiazepines and L-Dopa (Utz et al., 2010)^9^. Pregnancy is not a strict contraindication in tDCS research (DaSilva et al., 2011); however, we suggest that women who are pregnant or suspect they may be pregnant should be excluded as a matter of precaution. Participants with latent psychiatric illness (indicated by first degree relatives suffering from a psychotic disorder) should also be excluded (Meiron and Lavidor, 2013).

While there is nothing unique to military situations which require different safety guidelines to those used in therapeutic stimulation or cognitive enhancement of civilian populations, a different focus is required during screening processes. Conditions that are more prevalent in military personnel, such as post-traumatic stress disorder (Armenta et al., 2018), a history of addiction (Kelsall et al., 2015), or having metal implanted in the body (e.g., shrapnel; Gawande, 2004), should explicitly be stated and screened for. It should be noted that particular mental disorders are more prevalent in military personnel than in the wider civilian population. For example, a recent study found that 22% of Australian Defence Force members met ICD-10 (Tenth Revision of the International Statistical Classification of Diseases and Related Health Problems; World Health Organization, 1992) criteria for diagnosis of a mental health disorder at some point within the preceding 12 months period (van Hooff et al., 2014). Additionally, there is evidence that military personnel often do not disclose mental health issues due to perceptions of stigmatisation and career damage (e.g., Rüsch et al., 2017), so additional practical steps may be required in the military screening process. This would likely need to include a mechanism for impartial participant selection, such as encouraging potential stimulation recipients to consult with non-defence affiliated general practitioners, and acting on their eligibility recommendations without requiring justification or explanation (similar to some civilian pre-employment medical assessment procedures).

Finally, the risk of self-administration by defence personnel could be reduced through proactivity on the part of defence organisations. If tDCS were to be an area of active inquiry, military personnel could be involved in formal research rather than self-experimentation. If military research ultimately demonstrates that tDCS enhancement is worthwhile, the technology could then be implemented and administered safely, effectively, and ethically. If, however, it is concluded that the risks are too high and/or the benefits too variable, the informed position specific to military use will likely reduce (if not end) the temptation for private use by personnel. Either way, it would be beneficial for military organisations to have a concrete position on tDCS based on military-specific research.

## Future Prospects

A number of significant gaps remain in the tDCS body of literature in areas relevant to military use. Despite the considerable volume of literature indicating the potential of tDCS to enhance performance in areas relevant to military organisations, the case for its utility is somewhat diminished by the fact that it is currently unclear how much of the literature can be generalised into military situations. For tDCS to be truly beneficial, more research needs to be conducted investigating the applied aspects of using tDCS in military environments. This can be broken down into five main areas; inter-individual differences, the generalisability of research involving general populations to military personnel, whether the effects sizes of tDCS are large enough to make any practical difference to military capability and mission outcomes, the generalisability of laboratory based research into operational environments, and how tDCS could fit into established military personnel enhancement programmes.

The effects of tDCS are subject to a high amount of inter-individual variability, so stimulation of identical brain regions (via presumed locations under the skull bone) can cause different effects for different people or in different situations. While some authors have noted the requirement of relatively predictable and repeatable outcomes for the majority of stimulated individuals to justify tDCS as a treatment device in clinical settings (e.g., de Berker et al., 2013; Wiethoff et al., 2014), this requirement is arguably even more important in the military. Given that military situations often have life or death consequences, mean (average) group-level improvements are insufficient where the performance of some warfighters is reduced. Aside from the ethical responsibility to ensure that warfighters are not impeded during high risk situations, the overall effectiveness of the military team could also be diminished where a capability reduction of a single member encumbers the rest of the team. There is, therefore, a strong argument that the ideal goal should be for everyone’s performance to increase, with a minimum requirement that no one goes backward. Consequently, military tDCS use should be tailored to individual personnel.

Individually tailored stimulation is likely to be very difficult to implement. The effects of stimulation are affected by many factors and there is currently no standard process for calculating the likely response to stimulation to optimise target effects for any specific individual. The benefits of stimulation are not uniform across any given montage/protocol, with outcomes dependent on complex interactions between stimulation configurations and inter-individual differences in anatomical and cognitive characteristics (Sarkar et al., 2014). A non-exhaustive list of factors that can influence the effects of tDCS on any given individual is presented in Table 4.

**Table 4:** Variables known to affect the cognitive, motor, and behavioural outcomes of tDCS (derived in part from Li et al., 2015, pp. 9–14).

Factor	Reference
Age	Flöel et al., 2012; Fujiyama et al., 2014; Heise et al., 2014; Antonenko et al., 2018
Baseline task ability	Tseng et al., 2012; Learmonth et al., 2015
Bodyweight	Truong et al., 2013
Clinical predictors^1^	D’Urso et al., 2017
Cognitive load	Meiron and Lavidor, 2013
Cortical electric field	Laakso et al., 2015
Current strength	Teo et al., 2011; Batsikadze et al., 2013; Benwell et al., 2015
Distance between stimulation electrodes	Bikson et al., 2010; Moliadze et al., 2010
Duration of stimulation	Vignaud et al., 2018
Education	Berryhill and Jones, 2012
Electrode polarity	Jacobson et al., 2012
Electrode site	See examples in Tables 1, 2
Electrode size	Datta et al., 2009; Ho et al., 2016
Electrode type	Marshall et al., 2004
Extra-axial cerebrospinal fluid spaces	Beauchamp et al., 2011
Gender	Boggio et al., 2009; Meiron and Lavidor, 2013
Genetic constitution	Stephens et al., 2017
Head size^2^	Looi and Kadosh, 2015
Initial activation state of the stimulated network	Antal et al., 2007
Intensity of the stimulation	Iyer et al., 2005
Neurobiological differences	Giordano et al., 2017
Personality	Peña-Gómez et al., 2011
Pharmacological substance presence	Stagg and Nitsche, 2011
Psychological/cognitive traits	Dockery et al., 2009; Sarkar et al., 2014
Resting functional connectivity	Fischer et al., 2017
Stimulation programme pattern (e.g., repeated exposure, inter-stimulation interval timing, etc.)	Monte-Silva et al., 2010; Lin et al., 2011; Alonzo et al., 2012; Paneri et al., 2016
Task type	Andrews et al., 2011
Timing	Javadi and Cheng, 2012

*^*1*^For example: cognitive disturbance, retardation, anxiety, and somatisation.*

*^*2*^Skull size is highly correlated to age, and its effect on stimulation outcomes could be broken down further into related variables such as the ratio of grey to white matter in the brain and the thickness of the skull wall (Kessler et al., 2012).*

As well as having a large number of variables to consider, the difficulty of predicting an individual’s response is further amplified by the fact that many of the variables interact in complex ways. The majority of relationships between factors and the enhancement (or diminution) effects are neither linear nor monotonic (Laakso et al., 2015). For example, even the basic notion that activity tends to increase under the anodal electrode and decrease (or remain stable) under the cathodal electrode depends on the targeted brain region (Jacobson et al., 2012). Additionally, the effects can sometimes be completely reversed by factors like current strength (Batsikadze et al., 2013), gender (Meiron and Lavidor, 2013), or by complex interactions between variables (e.g., polarity × current strength × baseline task performance level; Benwell et al., 2015).

We therefore argue that delivering stimulation to individual military personnel based on evidence of group-level effects may be inappropriate, and any stimulation programme would be better delivered in a way that ensures efficacy. However, factoring in such a large number of complex interacting variables for each individual is incredibly problematic, and there is a strong likelihood that there are other influencing variables that have yet to be realised given that very little research has been conducted on the variability of effects. One potential approach is to simply perform tDCS on participants during an assessment session, and then divide participants into groups based on their level of activation (e.g., Wiethoff et al., 2014; López-Alonso et al., 2015). Generally, around three quarters of participants who are identified as responding positively in an initial session are likely to respond likewise in a later session. For example, López-Alonso et al. (2015) found that of the 60% of participants identified as responders in an initial session, 78% responded correspondingly in the second stimulation session. Although the authors concluded that cortical excitability responses in an initial session can reliably predict the responses of 69%^10^ of participants in a subsequent session (López-Alonso et al., 2015), this kind of strategy is not sufficient to predict the effects of tDCS for all personnel in all situations. Response patterns to tDCS are not represented bimodally (Fujiyama et al., 2014), so a more nuanced method than dichotomous splitting is required.

Other researchers have adopted more neurological focused approaches, using neuroimaging and modelling techniques to predict current flow based on individuals’ unique physiology/anatomy. These techniques include magnetoencephalography (Garcia-Cossio et al., 2016), electroencephalography (Schestatsky et al., 2013), functional magnetic resonance imaging based neuronavigation (Looi and Kadosh, 2015), surface-based registration^11^ processing of electric fields (Laakso et al., 2015), human head simulations (Datta et al., 2011), and dynamic causal modelling^12^ of neural network activity (de Berker et al., 2013). While this research has demonstrated that neuroimaging based simulations can successfully be used to better understand individual differences and optimise tDCS montages for individual participants based on brain current flow predications, practical utilisation of such techniques appear to be prohibitively difficult and time consuming (Davis et al., 2013). Many of the mechanisms involved in the effects of tDCS on brain excitability remain inadequately understood or disputed. Further studies are necessary to elucidate the mode of action of tDCS and determine the best paradigm of stimulation depending on the goals (Roche et al., 2015). Predicting the effects of tDCS can therefore only be so accurate before the physiological mechanisms producing the enhancements are adequately understood. Therefore, this is an area where more research is required, both in terms of specific tDCS neuroimaging research, and fundamental research towards developing a more comprehensive neurophysiological understanding.

Another required area of investigation is to establish the validity of the known effects of tDCS in the military context. Although the question of the real-world applicability of research conducted in artificial laboratory environments is by no means unique to tDCS or the military, the issue is more pronounced in military environments where warfighters experience extreme conditions such as stress, fatigue, and neurophysiological effects associated with exposure to danger (Canli et al., 2007). This is important because the effects of tDCS can be directly influenced by the state of brain activation at the time of stimulation (Canli et al., 2007; Levasseur-Moreau et al., 2013). For the general body of tDCS literature to be relevant to the military, the effects of tDCS need to be tested under conditions closely mimicking real-world military situations so the impact of external variables (such as stressful and high fatigue situations) can be better understood. It is strongly possible that a set of military tasking can be identified in which tDCS should not be used because of the likelihood of negative unintended consequences. In particular, it is the view of the current authors that the use of tDCS in situations likely to trigger strong emotional responses (such as those involving deadly force) is inappropriate until more is known about the interactions between environmental stressors, individual differences, and the effects of stimulation.

Another issue that needs to be considered is whether enhancement effects are large enough to have any meaningful impact (Levasseur-Moreau et al., 2013). This problem is not unique to tDCS research, as the suitability of emphasising statistical significance over effect sizes has been questioned for some time (e.g., Carver, 1978). However, it is particularly relevant in the context of examining technology with inherent risks. tDCS research has typically focused on general population enhancement and clinical population rehabilitation, and few studies directly address whether the effect sizes are large enough to be valuable for military purposes. However, there is some evidence suggesting that particular areas of interest can indeed be affected by tDCS in a meaningful way. Consider the following example from Levasseur-Moreau et al. (2013, p. 8):

“Pascual-Leone et al. (2012) estimated a mean reduction of 32 ms from studies using NIBS [non-invasive brain stimulation] to improve motor RT [reaction times]. In the specific context of speed shooting performances, ∼13 ms would be the difference between elite and rookie police officers (Vickers and Lewinski, 2012). Therefore, an improvement of 32 ms may make a vital difference in the context of a one-on-one gunfight or during aircraft combat (dogfight).”

However, it remains unclear what effect pre-existing skill level has on the level of benefit gained through tDCS. Given that other forms of enhancement (such as training) are subject to diminishing returns in levels of improvement, it is likely that a similar tapering is present in tDCS enhancement. Members of elite level Special Forces units, for example, may not experience the same level of improvement in their already highly refined skill set. Further research is required to confirm the model of diminishing gains, and determine the situations in which the enhancement effects of tDCS are large enough to have practical significance outweighing potential costs and risks.

Finally, it is worth noting that many of the effects elicited by tDCS can be achieved through other enhancement strategies, such as cognitive training (e.g., Biggs et al., 2015), fitness interventions (e.g., Hillman et al., 2008), meditation (e.g., Lutz et al., 2008), mindfulness training (e.g., Zeidan et al., 2010), nutrition (e.g., Burkhalter and Hillman, 2011), and video game training (e.g., Green and Bavelier, 2003). Many of these methods are already being utilised in existing military training programmes and interventions (e.g., Burton et al., 2011; Mead, 2013; Aidman, 2017). For tDCS to be valuable in the context of military enhancement, it must produce positive effects that are superior, additive, multiplicative, or associated with less adverse events than existing enhancement methods. Given that the mechanism of tDCS appears to alter resting membrane potential rather than directly producing action potentials, tDCS may be more beneficial in the complementation of existing methods rather than their replacement. While very little specific research has been conducted in this area, there is some evidence that tDCS can produce better outcomes than certain types of training (e.g., motor training; Pan et al., 2017), and that tDCS can increase the efficacy of cognitive training programmes in areas like dieting self-discipline (Forcano et al., 2018). Additional research establishing the interaction effects between tDCS and current military enhancement interventions is required before the efficacious delivery of tDCS can be ensured.

## Conclusion

The aim of this paper was to examine whether military tDCS use can be efficacious and ethical in military settings. Our assessment is that tDCS offers a number of cognitive, motor, and perceptual enhancement opportunities which could provide value in military situations like training and operations. There is potential scope for use in a number of key areas that directly affect practical battlefield advantage and survivability, such as deceptive capabilities, risk-taking, threat detection, perception, and physiological improvement. Additionally, tDCS has the potential to improve command and control decision making by enhancing aspects of cognition, such as planning, problem solving, memory, and probabilistic assessment.

As well as benefits, tDCS poses a number of risks that need to be considered, and in some cases addressed by military organisations. Overall, it is the opinion of the current authors that the safety concerns are manageable, and the risks moderate. The technique has been tested on thousands of subjects, with most studies reporting only temporary minor adverse effects such as itching, tingling, and headaches. The most severe adverse consequence of tDCS reported to date was abrasions at the locations of the electrodes (which can be mitigated through the application of saline soaked sponges enclosing conductive rubber electrodes). The intensity and rate of occurrence of adverse events are so low that some effects (such as headaches) are reported in similar rates between sham and active stimulation. Assuming that the equipment used can reliably deliver the current evenly and steadily (i.e., using medically certified equipment), the device is well-maintained, appropriate safety guidelines specific to the military are in place, and systematic screening is conducted to ensure that stimulation is performed only on suitable personnel, the risk of experiencing adverse effects appears to be no greater in military populations than in general populations.

Given the potential of tDCS to increase warfighter capabilities in support of command and control on a temporary, at needs basis, we submit that the potential benefits of tDCS have the potential to outweigh the limited and mitigable risks. In situations where tDCS can be used for the purpose of completing military objectives safely and where a marginal increase in performance can mean the difference between life and death, consequentialist arguments in support of military tDCS enhancement are compelling. However, this assumes that specific desired effects can be reliably delivered to individual warfighters. The effects are neither normally distributed nor even multimodally distributed, so there is a requirement to go beyond group-level findings that fail to account for the large number of inter-individual and environmental variables that affect the outcomes of stimulation.

Despite the potential of military tDCS application, there are a number of other open questions that need to be addressed before efficacious and ethical military stimulation is possible. It is unclear how applicable the current body of literature is in areas of military application. Research needs to be conducted investigating how specific effects of tDCS can be generalised across civilian and military populations given environmental factors and individual differences. While efficacious stimulation appears feasible, more research is required before tDCS can have a practical and substantial impact on capability and mission outcomes in the context of the current military training and enhancement landscape. As well as these empirical issues, a number of ethical questions require attention. It was not the purpose of this paper to take normative ethical stances beyond any apparent utilitarian conclusions that emerge from discussion of benefits and risk. Consequently, other ethical questions like the moral responsibility of warfighters’ actions while experiencing the effects of tDCS, and how traditional bioethical principles (such as respect for persons and justice) can be maintained, need to be considered separately.

As DIY tDCS becomes increasingly inexpensive and popular, the risk of military personnel experimenting with potentially dangerous devices or inadvertently using counterproductive montages increases. Given the pressing risks associated with self-administration, it would be preferable for military organisations to play a proactive role in addressing the remaining empirical and philosophical questions. If a military-based research programme were to conclude that tDCS is unable to provide genuine warfighting advantage, the temptation for riskier informal use by military personnel would be reduced (if not ended). If, however, the remaining issues (such as inter-individual variation) are adequately resolved, tDCS could then be administered in a controlled way that ensures safe, ethical, reliable, and efficacious delivery.

Overall, tDCS is a safe technique which may have potential for military application. Prospective benefits outweigh the safety risks when tDCS is conducted within established limits, and ethical concerns can be addressed through planning and policy. tDCS is acceptably safe when used in controlled conditions, and while additional research is required in a number of areas, tDCS has the potential to be valuable to military organisations once these issues are addressed and appropriate guidelines are enacted. While it is the view of the current authors that the state of the literature has yet to reach the point where ethical and efficacious delivery is possible in military settings, tDCS is presently fertile ground for military specific research.

## Author Contributions

SD was responsible for writing and preparation of the manuscript. SD and GS were jointly responsible for conceptualisation (scope, ideas, and substance) and editing (including critical review and revision).

## Conflict of Interest Statement

The authors declare that the research was conducted in the absence of any commercial or financial relationships that could be construed as a potential conflict of interest.

## Footnotes

1 The term “non-invasive" is used in the sense that no physical incision or insertion is made.

2 The Prussian general and theorist Carl von Clausewitz, for example, wrote on the “friction of war,” where “everything is very simple in war, but the simplest thing is difficult” (von Clausewitz, 1989, p. 119).

3 Streufert and Streufert (1968) argue that military situations often have an optimal level of risk which should ideally be matched to personnel with complementary risk taking propensities. Directly adjusting risk-taking behaviour could assist in this calibration (albeit while raising ethical issues that are beyond the scope of the present paper).

4 Indicated by a 14–34% decrease in the dopamine antagonist [^11^C]raclopride (Fonteneau et al., 2018).

5 Indirect in the sense that the dopaminergic affects are mediated through sensory organs (Heinz et al., 2012). For example, exercise (Hattori et al., 1994) and music consumption (Suzuki et al., 2008) can indirectly increase dopamine levels by approximately 50–100%.

6 Such as cocaine and amphetamine, which have been found to stimulate dopamine in the nucleus accumbens by up to 1000%, respectively (Di Chiara and Imperato, 1988).

7 Although tDCS does not involve incision or insertion, the technique is invasive in the sense that current spreads into adjacent brain tissue. This has led some authors (e.g., Davis et al., 2013) to contend that the term “non-invasive brain stimulation” may be inappropriate.

8 For a visual demonstration of tDCS procedure, see the online version of the article available at http://www.jove.com/video/2744/electrode-positioning-montage-transcranial-direct-current.

9 A specific list of drugs that interact with transcranial magnetic stimulation (TMS) is given by Rossi et al. (2009), and it is reasonable to assume that the same exclusion list is appropriate for tDCS studies (Davis et al., 2013).

10 This is an overall figure that also includes first session non-responders (which are significantly less reliable, with only 56% continuing not to respond in the second session).

11 Surface-based registration uses a spherical surface-based coordinate system which maps individuals’ brains based on structural and functional features (Fischl et al., 1999).

12 Dynamic causal modelling is a method for modelling connectivity in distributed neuronal response networks (Friston et al., 2003).
